# A Giant Vallecular Cyst in an Adult: A Case Report

**DOI:** 10.7759/cureus.64254

**Published:** 2024-07-10

**Authors:** Jasleen Kaur, Sagar S Gaurkar, Prasad T Deshmukh

**Affiliations:** 1 Department of Otolaryngology - Head and Neck Surgery, Jawaharlal Nehru Medical College, Datta Meghe Institute of Higher Education and Research, Wardha, IND

**Keywords:** vallecular cyst, tracheostomy, video-assisted laryngoscopy, marsupialization, dysphagia

## Abstract

Vallecular cysts (VCs) are rare benign lesions arising from the obstruction of mucous gland ducts. VCs are uncommon anomalies found in both pediatric and adult populations. They are also known as mucous-retention cysts, preepiglottic cysts, ductal cysts, base-of-tongue cysts, and epiglottis cysts. VCs are often asymptomatic in adults and may present with nonspecific symptoms such as globus sensation, voice changes, dysphagia, hoarseness, or airway obstruction when symptomatic. This case report details a rare occurrence of a giant VC in an adult male, emphasizing the diagnostic approach and surgical management and highlighting the importance of managing the airway in such cases and the advantages of endoscopic procedures.

## Introduction

Vallecular cysts (VCs) comprise approximately 5% of benign laryngeal lesions. Among all laryngeal cysts, VCs represent between 10.5% and 20.1% [1,2]. The first documented case of a VC was reported in 1881 [3]. While small laryngeal cysts may remain asymptomatic, larger ones can lead to serious complications [4]. In infants, these may include poor feeding, life-threatening airway obstruction, and failure to thrive. In adults, symptoms may encompass voice changes, dysphagia, hoarseness, and airway obstruction [4]. Nasopharyngolaryngoscopy is essential to diagnose and assess the extent of the cyst. Treatment options vary and may include cyst aspiration, marsupialization, deroofing of the cyst wall, and complete excision [3].

We present a 62-year-old male who presented with difficulty swallowing solids due to a large VC. The cyst was successfully managed through endoscopic marsupialization, leading to the complete resolution of symptoms without recurrence at a six-month follow-up. This case report highlights the importance of proper airway management and the effectiveness of endoscopic approaches in treating massive VCs in adults.

## Case presentation

**Figure 1:**
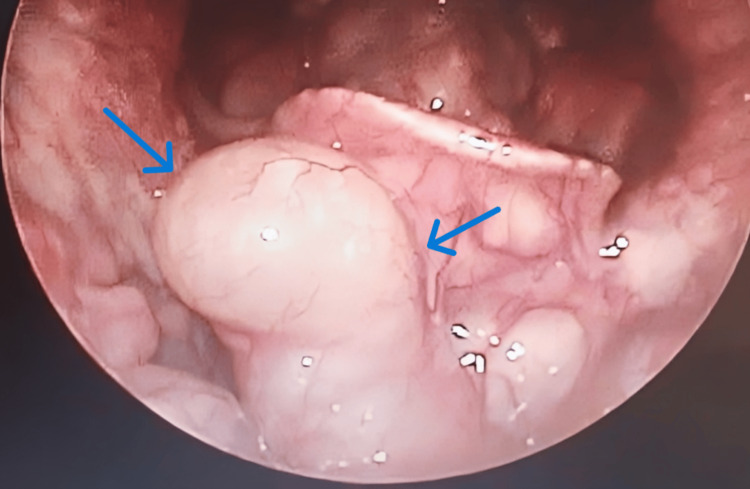
Video laryngoscopy revealed a whitish, cystic, smooth-surfaced mass, measuring approximately 3 x 4 cm, arising from the right aspect of the vallecula, and covering the lingual surface of the epiglottis (blue arrows)

A 62-year-old male visited the otorhinolaryngologist at a tertiary-level rural hospital in central India with complaints of difficulty swallowing solids. There was no history of recent weight loss, night sweats, loss of appetite, fever, infections, surgeries, hospitalizations, similar prior episodes, allergies, or any pertinent medical or family history. Routine ear, nose, and throat (ENT) clinical examinations were performed. Video-directed laryngoscopy (VDL) (Figure 1) revealed a whitish, cystic, smooth-surfaced mass, nonpulsating, noncongested, measuring approximately 3 x 4 cm, arising from the right aspect of the vallecula, displacing the epiglottis posteriorly, and covering its lingual surface. Small pseudocysts were also seen. Although his airway was compromised, he did not report any difficulty or noisy breathing, but his vocal cords were not visible. There was no lymphadenopathy in the neck, and the other physical examinations were unremarkable. 

**Figure 2:**
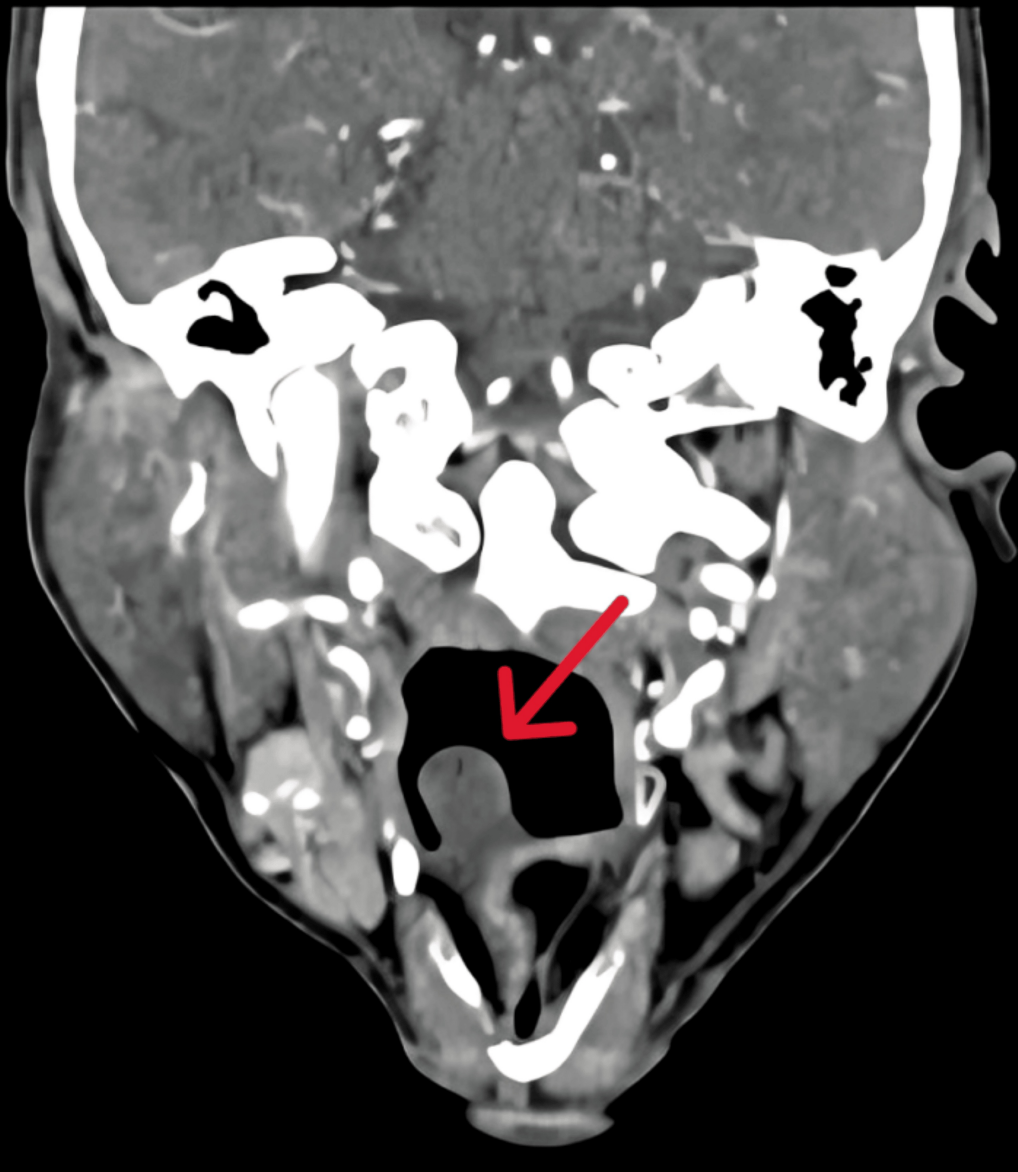
Computed tomography of the neck, where the coronal cuts show the cyst arising from vallecula (red arrow)

**Figure 3:**
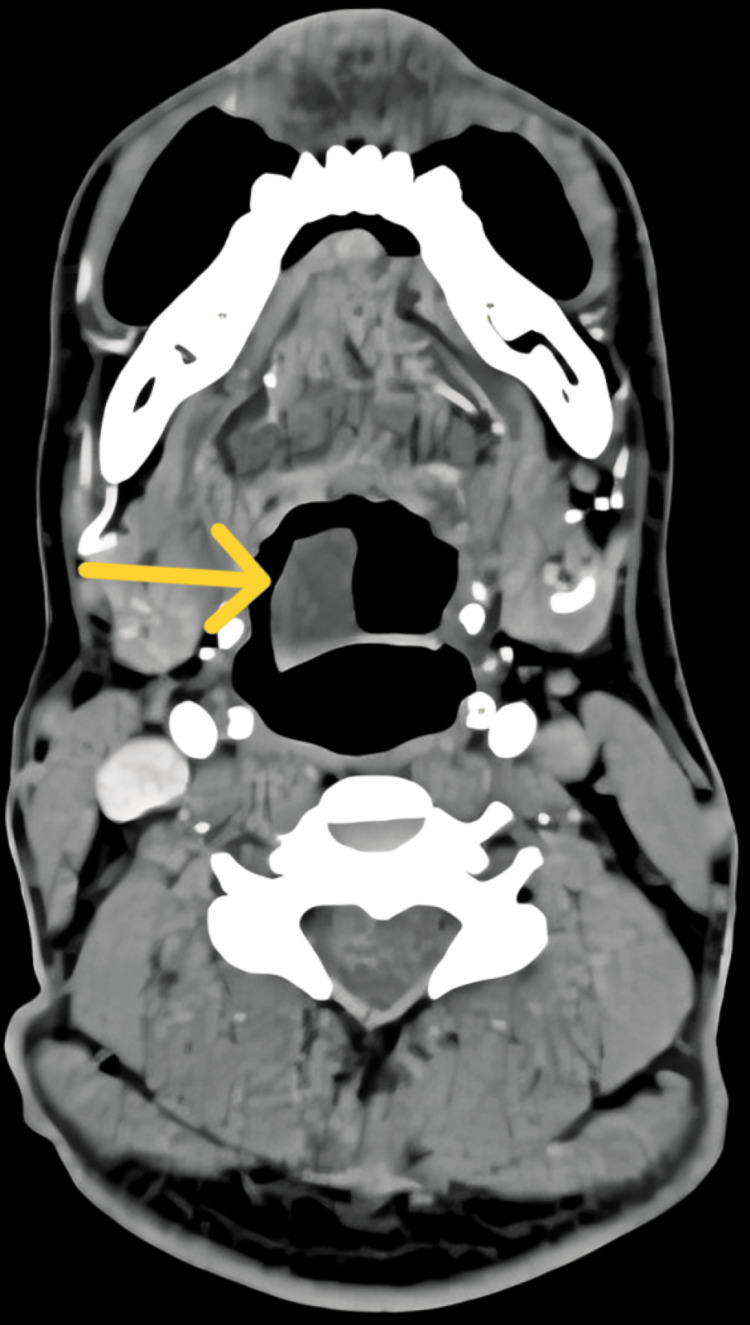
Computed tomography of the neck, where the axial cuts show the cyst arising from vallecula (yellow arrow)

A computed tomography scan is performed in cases of VCs to provide detailed images of the neck structures, allowing for precise assessment of the cyst's size, shape, and location. This imaging helps differentiate the cyst from other potential masses or lesions, such as tumors or abscesses, ensuring accurate diagnosis. It also aids in surgical planning by revealing the cyst's relationship to critical structures like the airway, blood vessels, and nerves. A computed tomography scan of the neck showed a well-defined cystic lesion on the right side of the vallecula measuring 3.7 x 2.6 cm (Figures 2, 3). Thyroid screening was normal. The patient was counseled about the potential need for emergency tracheostomy due to the anticipated difficulty of intubation.

Under general anesthesia, direct laryngoscopy confirmed the VDL findings. Partial aspiration of the cyst's contents was performed, revealing its origin and the pressure-induced displacement of the right side of the epiglottis. An incision was made over the cyst, and a thick whitish discharge was drained. The cyst wall was removed and sent for histopathological evaluation. Marsupialization of the cyst was performed, hemostasis was achieved, and the patient was observed overnight. He was advised to rest his voice and was discharged without complications the following day. His voice quality was normal, and his dysphagia gradually resolved. Histopathology confirmed a benign cyst. No recurrence was noted at the six-month follow-up.

## Discussion

Laryngeal cysts are classified into ductal and saccular types. Ductal cysts result from the obstruction and retention of mucus in the submucosal gland ducts, whereas saccular cysts develop from the saccule and may extend into the ventricle. Approximately 75% of laryngeal cysts are ductal. VCs commonly occur on the lingual surface of the epiglottis. They can occur at any age and are generally sporadic in nature [5].

VCs are believed to exhibit a bimodal presentation. Two main hypotheses explain their pathogenesis: one proposes that VCs result from ductal obstruction of mucous glands, whereas the other suggests they stem from an embryological malformation. The bimodal age distribution of VCs indicates that there may be two different clinical forms, with adult and pediatric VCs potentially originating from different underlying causes [6,7]. In a three-week-old embryo, the respiratory system begins to form as an endodermal outgrowth from the ventral wall of the foregut, just below the hypobranchial eminence. This outgrowth, the respiratory primordium, is initially separated from the dorsal portion, which becomes the esophagus, except at the entrance to the larynx, maintaining a connection to the foregut through the laryngeal orifice. Due to the close proximity of the primitive gut and the pharyngeal arches (which contain the developing tongue), parts of the gut can become misplaced in the developing tongue. These displaced epithelial remnants are thought to contribute to the formation of cystic lesions, such as VCs, in the tongue [8,9]. In contrast, VCs are believed to form in adults due to the obstruction of the submucosal minor salivary glands [9].

VCs are often asymptomatic, but when symptoms manifest, they can include stridor, cough, dysphonia, foreign body sensation, hoarseness, and dysphagia. Infection of the cyst can cause swelling and inflammation in nearby structures. Diagnosis typically involves indirect, direct, or flexible laryngoscopies. In our case, a thyroid screening was also conducted to rule out a lingual thyroid or thyroglossal cyst. Immediate intervention is necessary for patients experiencing severe respiratory distress or significant airway obstruction. Immediate management options include a needle cricothyrotomy or emergency cricothyrotomy with a trocar [6]. While cyst contents can sometimes be aspirated, this is not feasible for mucoid cysts. A ruptured cyst can make it difficult to visualize the vocal cords and increase the risk of aspiration. Histologically, the cyst is lined with respiratory epithelium containing mucous glands and is externally covered by squamous epithelium [7].

The differential diagnosis for lesions in the tongue base or vallecular region includes hemangioma, cystic hygroma, teratoma, hamartoma, dermoid cyst, lymphangioma, thyroglossal duct cyst, and thyroid remnant cyst [7].

Inhalational induction followed by orotracheal intubation is the preferred method in pediatric patients. When the patient cannot be intubated or ventilated by mask, a "cannot intubate, cannot ventilate" scenario arises, necessitating immediate life-saving rescue maneuvers. This was not the case for our patient; we promptly called in a more experienced anesthetist for intubation [8]. Treatment options include open surgery, endoscopic procedures (such as needle aspiration, marsupialization, and laser ablation), or a combination of these methods. Open procedures have disadvantages such as visible incision scars, longer anesthesia time, risk of superior laryngeal nerve injury, and extended hospital stay. Endoscopic procedures also have drawbacks, including limited exposure, high costs, the need for specialized equipment, difficulty in controlling bleeding, and the risk of thermal or airway injury. However, in our case, we did not experience these complications. The endoscopic approach had significant benefits, including the absence of an incision scar, shorter operation time, and quicker recovery [5,6]. According to Gutiérrez et al., when performed correctly, marsupialization is considered the definitive treatment for VCs. This procedure is safe and completely relieves upper airway obstruction in all patients. Recurrence of the cyst was noted in three cases where marsupialization was incomplete, necessitating a tracheostomy. Our findings also indicated no long-term recurrences in any patients when the procedure was done adequately [7]. As described in this case report, where the cyst obstructed the glottis view, aspiration was performed to alleviate pressure on the epiglottis and improve visualization. Following this, an incision was made over the cyst, and marsupialization was carried out to the level of the base of the tongue. Another emerging technique for VC excision is transoral laser-assisted surgery. Despite these challenges, our case did not experience these complications, highlighting the importance of careful preoperative planning and skilled surgical execution.

## Conclusions

In conclusion, VCs represent a rare yet manageable condition. This case report highlights the significance of including VCs in the differential diagnosis of dysphagia and acknowledges the complexities involved in their management. Endoscopic marsupialization emerged as a highly effective treatment option with minimal complications observed in our patient. Awareness of this uncommon condition is pivotal for ENT specialists, particularly in scenarios involving difficult intubation. Timely recognition and appropriate surgical intervention are crucial in achieving favorable patient outcomes, as evidenced by the resolution of symptoms and absence of recurrence in our case. Continued research and clinical vigilance are essential to further refine diagnostic approaches and optimize treatment strategies for VCs, ensuring improved patient care and outcomes in the future.
